# Supplementary material for: Novel Thin Film Nanocomposite Forward Osmosis Membranes Prepared by Organic Phase Controlled Interfacial Polymerization with Functional Multi-Walled Carbon Nanotubes
Source: Membranes (Basel). 2021 Jun 28;11(7):476. doi: 10.3390/membranes11070476 (PMC8304348; doi:10.3390/membranes11070476)
Supplement: Supplementary file 1 [file membranes-11-00476-s001.zip › membranes-1260765-supplementary.pdf]

Supporting Information

Novel thin film nanocomposite forward osmosis membranes prepared by organic phase controlled interfacial polymerization with functional multi-walled carbon nanotubes

Xu Zhang [1,§], Jiuhan Zheng [1,§], Lusheng Xu [1], Ming Yin [1], Guoliang Zhang [1,*], Wenqian Zhao [1], Zeyu Zhang [1], Chong Shen [2], Qin Meng [2,*,*]

[1] Center for Membrane and Water Science &Technology, Institute of Oceanic and Environmental Chemical Engineering, State Key Lab of Green Chemical Synthesis Technology, Zhejiang University of Technology, Hangzhou 310014, P.R.China

[2] College of Chemical and Biological Engineering, State Key Laboratory of Chemical Engineering, Zhejiang University, Zhejiang University, Hangzhou 310027, P.R.China

* Correspondence: guoliangz@zjut.edu.cn; Tel.: +86-571-88320863 (G.Z.); mengq@zju.edu.cn; Tel.: +86-571-87951227 (Q.M.)

* These authors contributed equally.

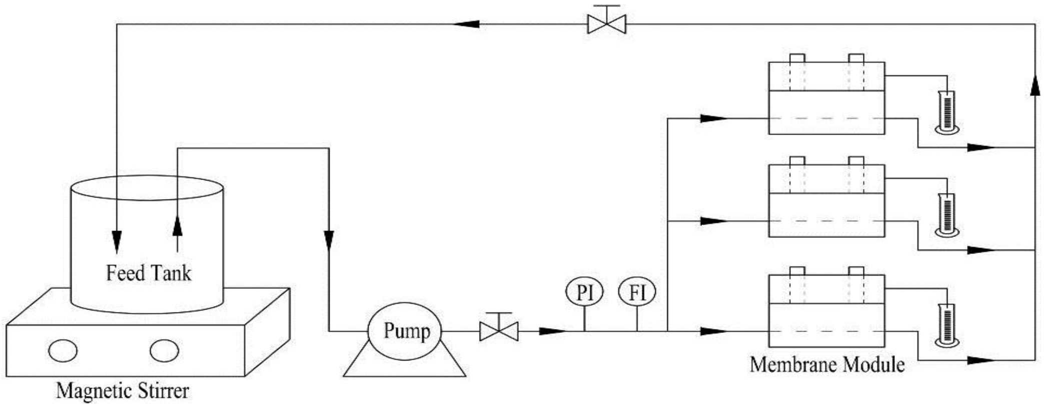

Figure S1. Schematic diagram of cross-flow RO system.

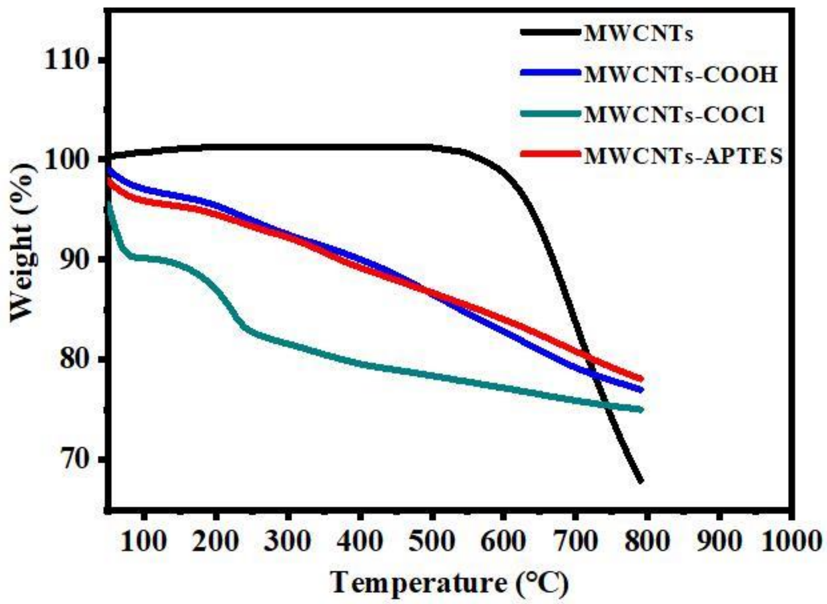

Figure S2. TGA curves of MWCNTs, MWCNTs-COOH, MWCNTs-COCl and MWCNTs-APTES.

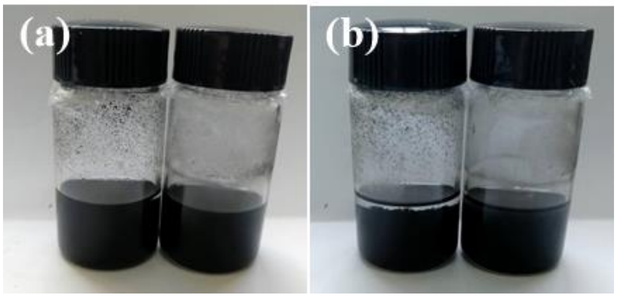

Figure S3. Optical photographs of MWCNTs and MWCNTs-APTES dispersion after keeping static for **(a)** 0 second and **(b)** 10 min. (left: MWCNTs, right: MWCNTs-APTES).

Table S1. Performance of TFN membrane compared with other CNTs-based FO membranes using DI water as feed solution.

Membrane	Draw solution	Membrane Orientation	J_w(LMH)	J_s(gMH)	J_s/J_w(gL)	Ref
TFN-aCN/AP	1 M NaCl	AL-FS	46.0	1.2	0.026	S1
M-T/CNT/PA	1 M NaCl	AL-DS	28.48	7.4	0.26	S2
		AL-FS	~18	~4.8	~0.27	
PET30-mCNT-HPAN30	1 M NaCl	AL-DS	43.5	11.1	0.255	S3
		AL-FS	29.0	9.4	0.32	
PVDF/SiO_2@MWNTs TFC	1 M NaCl	AL-FS	22.1	4.1	0.19	S4
TFN/SCNTs	1 M NaCl	AL-FS	29.9	~3.5	0.12	S5
TFC-CNT	2 M NaCl	AL-DS	31	6.2	0.2	S6
TFC MMM	0.6 M NaCl	ALFS	11.98	7.7	0.64	S7
0.5MWfT/M-P	1 M NaCl	ALFS	12.7	5.8	0.46	S8
Car-PES TFC	2 M NaCl	AL-DS	25.8	12.3	0.476	S9
		AL-FS	16.1	8.0	0.496	
TFN	1 M NaCl	ALDS	22.2	2.6	0.117	This work
		AL-FS	19.6	1.7	0.091	

Supplementary References

S1. Wang, D.; Li, J.; Gao, B.; Chen, Y.; Wang, Z. Triple-layered thin film nanocomposite membrane toward enhanced forward osmosis performance. *J. Membr. Sci.* **2021,** *620*, 118879.

S2. Zhang, M.M.; Jin, W.B.; Yang, F.L.; Duke, M.; Dong, Y.C.; Tang, C.Y.Y. Engineering a Nanocomposite Interlayer for a Novel Ceramic-Based Forward Osmosis Membrane with Enhanced Performance. *Environ. Sci. Technol.* **2020,** *54*, 7715-7724.

S3. Tsai, M.T.; Chung, L.H.; Lin, G.Y.; Chang, M.C.; Lee, C.Y.; Tai, N.H. Layered carbon nanotube/polyacrylonitrile thin-film composite membrane for forward osmosis application. *Sep. Purif. Technol.* **2020,** *241*, 116683.

S4. Zhang, X.; Shen, L.; Guan, C.Y.; Liu, C.X.; Lang, W.Z.; Wang, Y. Construction of SiO_2@MWNTs incorporated PVDF substrate for reducing internal concentration polarization in forward osmosis. *J. Membr. Sci.* **2018,** *564*, 328-341.

S5. Li, Y.H.; Zhao, Y.T.; Tian, E.L.; Ren, Y.W. Preparation and characterization of novel forward osmosis membrane incorporated with sulfonated carbon nanotubes. *Rsc Advances* **2018,** *8*, 41032-41039.

S6. Zhao, X.Z.; Li, J.; Liu, C.K. A novel TFC-type FO membrane with inserted sublayer of carbon nanotube networks exhibiting the improved separation performance. *Desalination* **2017,** *413*, 176-183.

S7. Choi, H.G.; Son, M.; Choi, H. Integrating seawater desalination and wastewater reclamation forward osmosis process using thin-film composite mixed matrix membrane with functionalized carbon nanotube blended polyethersulfone support layer. *Chemosphere* **2017,** *185*, 1181-1188.

S8. Morales-Torres, S.; Esteves, C.M.P.; Figueiredo, J.L.; Silva, A.M.T. Thin-film composite forward osmosis membranes based on polysulfone supports blended with nanostructured carbon materials. *J. Membr. Sci.* **2016,** *520*, 326-336.

S9. Liu, Q.; Qiu, G.L.; Zhou, Z.Z.; Li, J.G.; Amy, G.L.; Xie, J.P.; Lee, J.Y. An Effective Design of Electrically Conducting Thin-Film Composite (TFC) Membranes for Bio and Organic Fouling Control in Forward Osmosis (FO). *Environ. Sci. Technol.* **2016,** *50*, 10596-10605.
